# Photocrosslinking of cDNA Display Molecules with Their Target Proteins as a New Strategy for Peptide Selection

**DOI:** 10.3390/molecules25061472

**Published:** 2020-03-24

**Authors:** Takuya Terai, Tomoyuki Koike, Naoto Nemoto

**Affiliations:** Graduate School of Science and Engineering, Saitama University, 255 Shimo-Okubo, Sakura-ku, Saitama City, Saitama 338-8570, Japan

**Keywords:** directed evolution, in vitro selection, multimolecular crowding biosystems, cDNA display, photocrosslinker, peptide aptamer

## Abstract

Binding peptides for given target molecules are often selected in vitro during drug discovery and chemical biology research. Among several display technologies for this purpose, complementary DNA (cDNA) display (a covalent complex of a peptide and its encoding cDNA linked via a specially designed puromycin-conjugated DNA) is unique in terms of library size, chemical stability, and flexibility of modification. However, selection of cDNA display libraries often suffers from false positives derived from non-specific binding. Although rigorous washing is a straightforward solution, this also leads to the loss of specific binders with moderate affinity because the interaction is non-covalent. To address this issue, herein, we propose a method to covalently link cDNA display molecules with their target proteins using light irradiation. We designed a new puromycin DNA linker that contains a photocrosslinking nucleic acid and prepared cDNA display molecules using the linker. Target proteins were also labeled with a short single-stranded DNA that should transiently hybridize with the linker. Upon ultraviolet (UV) light irradiation, cDNA display molecules encoding correct peptide aptamers made stable crosslinked products with the target proteins in solution, while display molecules encoding control peptides did not. Although further optimization and improvement is necessary, the results pave the way for efficient selection of peptide aptamers in multimolecular crowding biosystems.

## 1. Introduction

**Figure 1:**
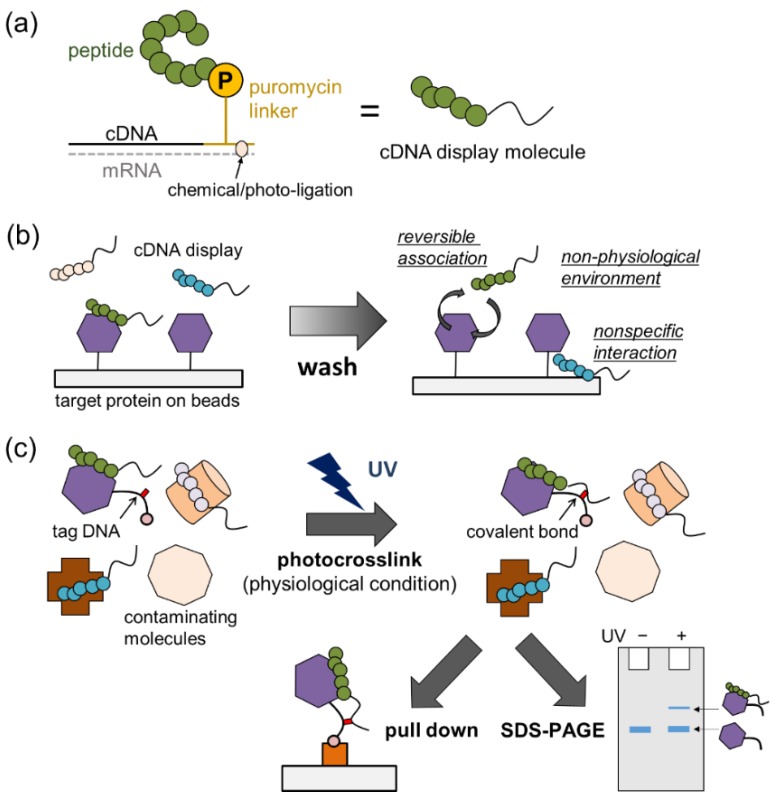
Photocrosslinking between complementary DNA (cDNA) display molecules and target proteins. (**a**) Schematic representation of a cDNA display molecule, where P indicates puromycin. (**b**) Representation of current selection scheme and its limitations. Because interactions between cDNA display molecules and target proteins are reversible, affinity molecules may be dissociated during washing. (**c**) New selection scheme proposed in this work. cDNA display molecules are incubated with a mixture of proteins in solution. Only the target protein is chemically conjugated with a tag DNA that contains a photocrosslinker (red square). After ultraviolet (UV) light irradiation, covalent linkage is selectively achieved, and the crosslinked product can be purified by affinity pull-down or electrophoresis.

Directed molecular evolution is a powerful technology to create polypeptides with desired properties. To evolve peptides, it is essential to chemically or physically link the peptides with the nucleic acids that encode them. Although in vivo systems, where each mutant gene is introduced to a single *Escherichia coli* (or yeast) cell, are quite useful for enzyme evolution [1,2,3] and antibody selection using the phage (or yeast surface) display technique [4,5,6], in vitro systems that do not rely on transformation have advantages due to their larger library size and higher selection speed. Several in vitro linkage methodologies, including ribosome display [7], messenger RNA (mRNA) display [8,9,10], and microbead display [11], were developed and used for peptide selection. Among them, complementary DNA (cDNA) display (Figure 1a) [12] is a promising technology to identify peptide aptamers or single-domain antibodies from a large library with high throughput. In cDNA display, a special DNA linker (Figure S1, Supplementary Materials) featuring puromycin (an antibiotic that is incorporated at the C-terminal of a nascent peptide in the A site of ribosomes) is ligated to an mRNA library, yielding an mRNA–linker complex. In vitro translation and mRNA–protein linkage is then performed, and the mRNA moiety is reverse-transcribed to make cDNA–protein conjugates. The formed cDNA display molecules are incubated with target-immobilized magnetic beads, and, after some washing steps, bound molecules are collected and their cDNA is amplified by polymerase chain reaction (PCR) (Figure S2, Supplementary Materials). Whereas reverse transcription is performed before selection in most modern mRNA display procedures [10], cDNA display is different in that the reverse transcription primer site is incorporated into the puromycin linker and, thus, the cDNA moiety is covalently linked to the peptide via the linker. Compared with other techniques, cDNA display has advantages in terms of library size (typically 10^12–13^) and stability under harsh conditions, such as organic solvents, strong acids and bases, and heat. We and others used cDNA display to identify peptides associated with proteins [12,13,14], lipid membranes [15], and small molecules [16,17]. 

However, both cDNA and mRNA display molecules have a practical drawback of nonspecific interaction with the solid phase (i.e., magnetic microbeads) and cationic/hydrophobic proteins [10]. To address this issue, negative selections using target-free control beads must be performed in parallel with or before the actual selection [12,13,14,16]. Unfortunately, according to our experience, the problem is solved only partially by this strategy, presumably due to the stochastic nature of nonspecific binding. Furthermore, adjustment of washing conditions is often tried to remove nonspecific or weak binders but still retain strong specific binders [14,16]. Whereas this seems to work intuitively, when the system contains only a few cDNA display molecules, each featuring specific binding peptides (with their *K*_d_ values in the middle nanomolar range), the probability is high that most will be lost during washing (Figure 1b). In addition, it is quite difficult to find suitable washing conditions to discriminate specific and nonspecific binders in practice. Another potential problem of conventional screening schemes is that the target molecule is immobilized on a solid, and the selection is performed in pure and artificial buffers that do not contain other biomolecules. These conditions should promote the survival of peptides that bind nonspecifically to many proteins and reduce the signal-to-noise ratio of the selection. 

In this work, to address the above problems, we propose a novel selection scheme using cDNA display (Figure 1c). We firstly hypothesized that one of the fundamental limitations of current affinity selections using cDNA (or mRNA) display lies in the non-covalent and reversible nature of their interaction with the target molecules. Then, selective conversion of the interaction between a cDNA display molecule and its target protein to a covalent bond should be an attractive solution to improve the signal-to-noise ratio of in vitro selections. Thus, we focused on a photocrosslinking nucleic acid that makes a covalent bond with a base in its complementary strand upon light irradiation [18]. Every cDNA display molecule has a fixed single-stranded DNA (ssDNA) moiety in its puromycin linker part, which can be used for hybridization. Moreover, the target protein can be chemically conjugated with a short ssDNA incorporating a photocrosslinker, hereafter called a tag DNA. When a library of cDNA display molecules is added to the mixture of the target protein labeled with a tag DNA and other contaminating molecules in solution, display molecules interact with their respective target molecules. Then, ultraviolet (UV) light is shone onto the sample to crosslink the puromycin linker DNA and the tag DNA. Because of its pseudo-intramolecular nature, crosslinking of the cDNA display that is bound to the target should be much more efficient than that of cDNA display molecules in free solution or those bound to contaminating molecules. The crosslinked complex can be purified by pull-down using another tag molecule conjugated to the target protein, such as biotin or poly adenine (A). It can also be checked and/or isolated by gel or capillary electrophoresis. 

Covalent conjugation of target proteins and small-molecular ligands was recently actively studied in the field of chemical biology, bioimaging, and drug discovery [19,20,21,22]. Chemical and photoaffinity labeling of DNA-encoded compounds [23,24] and DNA aptamers [25] to target proteins were reported to identify unknown targets or screen novel binders. Furthermore, Liu et al. reported a method called “interaction determination using unpurified proteins” to selectively amplify DNA sequences encoding the information of ligand–target pairs from a heterogenous mixture of DNA-encoded chemical library and proteins [26]. In their method, the target proteins were also tagged with short DNA that hybridized with DNA barcodes on the ligands. Although the idea of this study was partly inspired by these previous works, as far as we know, it is the first example of photocrosslinking cDNA or mRNA display molecules to target proteins, and the results are expected to be useful for future improvement of the selection strategy.

## 2. Results and Discussion

### 2.1. Model Study of Photocrosslinking

**Figure 2:**
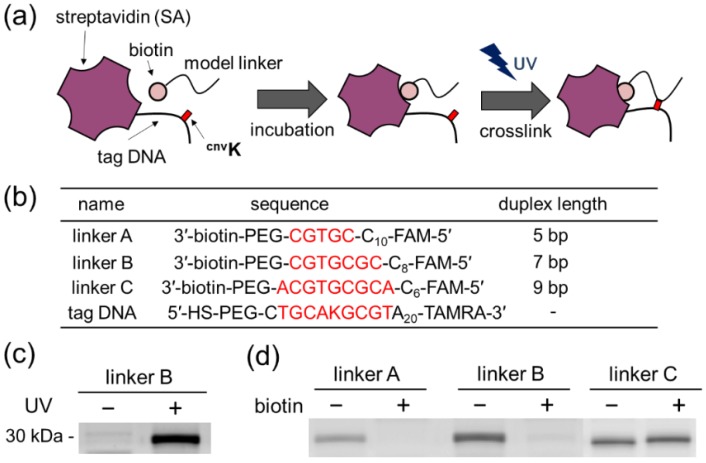
Ligand-dependent photocrosslinking of linker DNA to a model protein. (**a**) Schematic representation of the experiment. A tag DNA containing ^cnv^K was chemically conjugated to streptavidin (SA), and irradiation was performed after incubation with a biotin-labeled model linker DNA with or without a competitor ligand (free biotin). (**b**) Oligonucleotides used in this experiment. carboxyfluorescein (FAM) and tetramethylrhodamine carboxylic acid (TAMRA) are fluorescent molecules for detection. SH stands for thiol, and hybridized bases are marked in red. PEG indicates a polyethylene glycol (PEG) spacer 18. (**c**) Confirmation of photocrosslinking. SA-tagged DNA and a linker (duplex length = 7-mer) were mixed, and UV irradiation was performed before SDS-PAGE. The gel image was taken using a fluorescence imager with a FAM filter set. (**d**) Inhibition of crosslinking by free biotin. All samples were irradiated with UV light before analysis.

To selectively photocrosslink a tag DNA (a short ssDNA that is conjugated to the target protein) and the puromycin linker of a cDNA display molecule that encodes a binding peptide (see Figure 1c), the two DNA strands must be dynamically hybridized only when they are in close proximity. In other words, the melting temperature of the hybridization region should be close to the ambient temperature. At the same time, the hybridization length should not be too short to avoid nonspecific hybridization. Although we could find previous papers using six or seven bases as the duplex length for such purposes [27,28], we decided to set the length experimentally using the set-up analogous to our selection model. Here, streptavidin (SA) was chosen as a model target, and biotin was used as a model ligand (Figure 2a) because biotin-conjugated oligonucleotides are commercially available. For a photocrosslinking base, 3-cyanovinylcarbazole nucleoside (^cnv^K) [18] was selected, which induces an ultrafast DNA interstrand photocrosslinking reaction with a thymine (T) or uracil (U) base upon irradiation at 366 nm. As a tag DNA, 5ʹ-thiol-modified DNA containing ^cnv^K was synthesized (Figure 2b) and labeled with lysine residues of SA using *N*-(6-maleimidocaproyloxy)succinimide (EMCS) as a hetero-bifunctional chemical crosslinker. As is well known, this crosslinking strategy does not discriminate lysine residues on the protein surface, and the obtained labeled protein is heterogenous in terms of the number and position of labeled tag DNAs. Whereas it may be a potential limitation of our methodology, there could be some methods to circumvent this issue (see Section 4). Notably, ^cnv^K was previously incorporated into a puromycin linker and successfully used to conjugate mRNA and the linker [16]. Thus, its compatibility with the cDNA display technique is guaranteed. As a model puromycin linker encoding a peptide aptamer, three 3ʹ-biotin-modified DNA molecules were designed with different duplex lengths (five, seven, and nine bases, Figure 2b). For detection, the tag DNA and the linker DNA were labeled with tetramethylrhodamine carboxylic acid (TAMRA) and carboxyfluorescein (FAM) fluorescent dyes, respectively. 

The experimental scheme is shown in Figure 2a. SA labeled with tag DNA was mixed with a model linker and UV irradiation was performed in the presence and absence of excess free biotin. The samples were analyzed by SDS-PAGE containing 8 M urea, and the gel was visualized using a FAM filter set. As shown in Figure 2c (a larger gel image is shown in Figure S3, Supplementary Materials), the product of covalent conjugation was obtained only after UV irradiation at around 30 kDa, which agrees with the sum of molecular weights of monomer SA (~15 kDa), tag DNA (~9 kDa), and the linker (~6 kDa). For the linkers with 5- and 7-mer duplex lengths, the crosslinking was inhibited by free biotin for more than 90% (Figure 2d). Although the background crosslinking was higher for the 7-mer for about 1.5-fold, we thought the difference was small. Compared with the 5-mer duplex, the 7-mer duplex seemed to give better crosslinking yield over three-fold without a substantial increase in the yields of background reactions in the presence of biotin. In contrast, when the linker with 9-mer duplex length was used, free biotin did not suppress crosslinking at all, indicating that duplex formation (and subsequent photocrosslinking) was not dependent on protein–ligand interaction but rather driven by the high thermodynamic stability of the hybridization. These results suggested that photocrosslinking between peptide aptamers and target proteins should be feasible using appropriately designed tag DNA and a puromycin linker. In the following experiments, we used a 7-mer duplex length. 

### 2.2. Synthesis of Photocrosslinkable Puromycin Linker and Formation of cDNA Display

**Figure 3:**
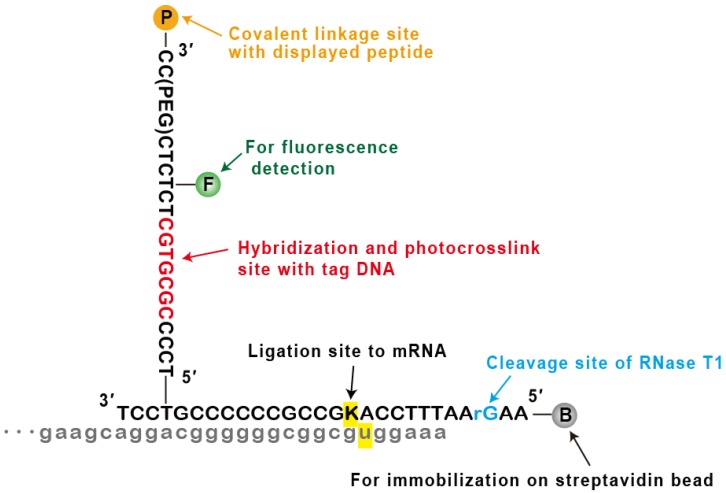
Structure of photocrosslinking (PC)-linker. P indicates puromycin, F indicates fluorescein, B indicates biotin, rG indicates guanine (not deoxyguanine), PEG indicates polyethylene glycol spacer 18, and K indicates ^cnv^K (the ligated bases are highlighted in yellow). For clarity, the 3′ terminal of messenger RNA (mRNA) is shown in gray. For more details, please refer to Section 3.

Next, we designed and synthesized a new puromycin linker, called photocrosslinking (PC)-linker, which could photocrosslink with the tag DNA. The 7-mer single-stranded part described above was introduced in the middle of the branched chain, or puromycin fragment (Figure 3, Table S1, Supplementary Materials). The main chain, or biotin fragment (Figure 3, Table S1, Supplementary Materials), was not changed from the previously developed linker (^cnv^K-rG linker) [16]. Both fragments were chemically synthesized by vendors and connected in-house according to the established protocol (Figure S4, Supplementary Materials; see Section 3 for details). 

The new PC-linker was then used for the preparation of the cDNA display. As a displayed peptide for model experiments, we chose SA-binding peptide (SBP), which was reported by Szostak et al. as a nanomolar binder for SA [29]. The DNA construct encoding SBP and a His_6_ tag (Figure S5, Supplementary Materials; the construct also had 5ʹ and 3ʹ untranslated regions) was transcribed in vitro and ligated to the PC-linker. The ligation product was translated in vitro, reverse-transcribed on magnetic beads, and purified using Ni beads to yield cDNA display molecules (see Section 3 for details). The efficiency of cDNA display formation from mRNA was about 10% (Figure S6, Supplementary Materials), which was not very different from the average efficiency of previous linkers (5%–25%). Using the PC-linker, we also prepared a cDNA display encoding the B domain of protein A (BDA) [30], which is a small protein domain that has an affinity for immunoglobulin G (IgG). 

### 2.3. Photocrosslinking of cDNA Display Molecules

**Figure 4:**
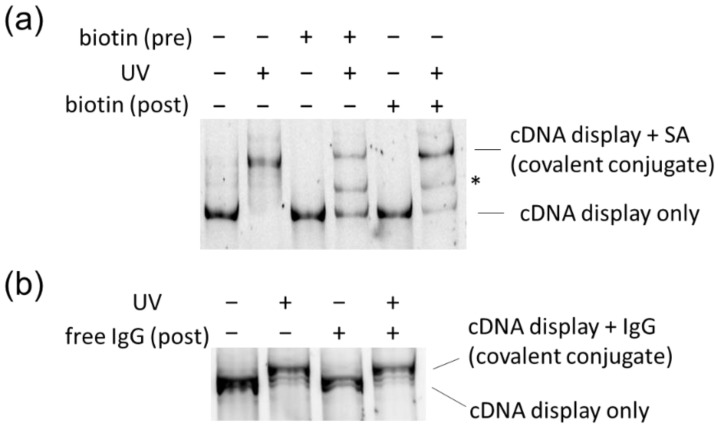
Affinity-dependent photocrosslinking between cDNA display molecules and target proteins. (**a**) Crosslinking of cDNA display encoding SA-binding peptide (SBP) and SA labeled with tag DNA. They were incubated with and without biotin, and UV irradiation was performed before PAGE analysis. For lanes 5 and 6, biotin was added after irradiation. The gel was visualized with FAM that was attached to the cDNA display. The asterisk (*) indicates an uncharacterized sub-band. (**b**) Crosslinking of cDNA display encoding the B domain of protein A (BDA) and immunoglobulin G (IgG). For lanes 3 and 4, free IgG was added after irradiation.

The prepared cDNA display molecules featuring the PC-linker were incubated with their respective target proteins (SA or IgG) that were conjugated with tag DNA. The incubation and UV irradiation were performed in phosphate-buffered saline (PBS) solution, and the photocrosslinking reaction was monitored by PAGE. For simpler characterization of bands, proteins were not denatured by SDS or heat in these experiments, because both target proteins were oligomers. The results of SBP–SA pairing are shown in Figure 4a. After UV irradiation, the band of the cDNA display encoding SBP was upshifted, suggesting that covalent crosslinking of the peptide and tetrameric SA protein occurred (compare lanes 1 and 2). Because excess SA (>10-fold) was added, we believe that multiple binding of cDNA display molecules to one SA molecule can be ignored here. Without UV, the noncovalent association of the polypeptide–DNA conjugates seemed to be weak enough to dissociate during PAGE. Notably, the crosslinking was significantly inhibited by pre-treatment of SA with free biotin (compare lanes 2 and 4), in line with the fact that SBP and biotin compete for the binding pocket of SA [29]. We also prepared a sample where biotin was added after UV irradiation (lanes 5 and 6). Here, most of the crosslinked product remained intact, further demonstrating the irreversible nature of crosslinking. There is also a detectable band corresponding to free cDNA display at lane 6, which might reflect the tetrameric nature of the SA molecule, high steric hindrance of the quaternary complex (SA, tag DNA, biotin, and cDNA display molecule), or incomplete photocrosslinking. So far, we do not understand the identity of the new band that appeared between the free and SA-bound cDNA display after irradiation (marked with an asterisk, lanes 4 and 6). Because the band was not observed in the absence of competing biotin (lane 2), it may represent a complex of cDNA display encoding SBP and the monomer (or dimer) of SA, which was somehow stabilized by biotin. As another control, we performed the same experiment using SA that was not modified with tag DNA (Figure S7, Supplementary Materials). As expected, an upshift of bands in response to UV irradiation was not observed under this condition, indicating that photocrosslinking was dependent on hybridization of tag DNA and the PC-linker. Notably, without tag DNA, most of the cDNA display molecules formed a strong noncovalent complex with SA, which was observed as an upshifted band even in the absence of UV irradiation (lane 1, Figure S7, Supplementary Materials). This suggested that the affinity of SA and SBP was weakened to some extent after modification of tag DNA on the protein. Steric hindrance and electrostatic repulsion between tag-DNA moiety and cDNA moiety would be other factors. Even so, the moderate interaction of SBP and labeled SA was successfully captured as a covalent complex after UV crosslinking (lane 2, Figure 4a). Therefore, as long as photocrosslink-based selection is performed, it would not be a serious problem.

We also performed crosslinking experiments between cDNA display molecules encoding BDA and IgG protein (Figure 4b). A UV-induced upshift of the display band was observed (compare lanes 1 and 2), and the shift was not inhibited by addition of free IgG after irradiation (lane 4). Taken together, these results indicated that it was feasible to photocrosslink cDNA display molecules and the target proteins using the PC-linker and tag DNA. 

### 2.4. Model Selection in the Presence of Contaminating Molecules

**Figure 5:**
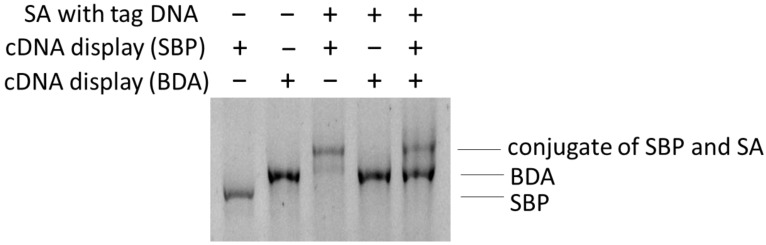
Model selection of binding peptides for SA. cDNA display molecules were incubated with SA modified with tag DNA in the presence of excess bovine serum albumin (BSA). After UV irradiation, samples were analyzed by PAGE. Although the cDNA display encoding BDA did not form a covalent crosslinked product with SA (compare lanes 2 and 4), the display encoding SBP did (compare lanes 1 and 3). When the two display molecules were mixed, only the SBP band showed upshifting, while that of BDA remained completely intact (lane 5). The two bands of lane 5 were dissected and analyzed by PCR.

Finally, as a model of photocrosslinking-based selection, we mixed SA (labeled with tag DNA) with cDNA display molecules encoding SBP and BDA to examine whether only the correct binder peptide (SBP, in this case) was photocrosslinked to the protein. Furthermore, to demonstrate the potential compatibility of the technique with selections in multimolecular crowding biosystems, an excess amount of contaminating protein, bovine serum albumin (BSA), was added to the samples. As shown in Figure 5, even under this condition, photocrosslinking of SA and SBP was confirmed (compare lanes 1 and 3). However, crosslinking of SA and BDA was not observed at all (lane 4), further demonstrating that the photocrosslinking reaction depended on peptide–target interaction. Notably, crosslinking of SBP and SA was not affected by the presence of non-binding cDNA display molecules (BDA, see lane 5) or a non-binding protein (BSA, all lanes). We also confirmed that by dissecting the band of the conjugate and PCR amplifications of DNA therein, genetic information of the binding peptide (SBP) was selectively obtained (Figure S8, Supplementary Materials). In Figure S8 (Supplementary Materials), some of the readers may recognize a faint band in lane 4, which corresponded to the contaminating DNA encoding BDA. We note, however, that its band intensity was low (less than 5% compared to the DNA of SBP) and that its appearance was mainly due to the mere technical difficulty of accurately dissecting the upshifted band under this electrophoresis condition. In short, these results collectively suggest the feasibility of the new selection scheme that we proposed in Figure 1c. 

## 3. Materials and Methods 

### 3.1. Reagents and Instruments

General chemicals were of the best grade available and supplied by Wako Pure Chemical Industries (Osaka, Japan). Chemicals for molecular biology experiments were obtained from Sigma and Wako Pure Chemical Industries. They were used without further purification. DNA oligos were synthesized by Eurofins Genomics and Hokkaido System Science (Sapporo, Japan). For water, MilliQ (Merck Millipore, Burlington, VT, USA) was used. 

PCR was performed with a Biometra TRIO48 thermal cycler. Unless otherwise stated, PrimeSTAR HS DNA polymerase (Takara Bio, Kusatsu, Japan) was used for PCR under the conditions recommended by the manufacturer, and DNA was purified using a FavorPrep PCR Clean-Up Mini Kit (Favorgen, Ping-Tung, Taiwan). For the primers and synthetic oligos, see Table S1 (Supplementary Materials). Gel images were taken with a Typhoon FLA9500 imager (GE Healthcare, Chicago, IL, USA). Unless otherwise stated, PAGE analyses of DNA and RNA were performed at 60 °C using gels containing 8 M urea, with 0.5× Tris-Borate-EDTA (TBE) as a running buffer. SDS-PAGE analyses of cDNA display molecules were performed at room temperature (r.t.) using Tris-HCl gels containing 8 M urea. 

### 3.2. Conjugation of Tag DNA to Proteins

Tag DNA with a 5ʹ-thiol group (5 nanomole, obtained from Hokkaido System Science) was reduced by incubation with dithiothreitol (DTT, 0.1 M) in 0.4 M sodium phosphate (100 μL, pH 9.0) at r.t. for 1 h. DTT was removed and the buffer was exchanged to sodium phosphate (20 mM, pH 7.2) with 30 mM NaCl using an NAP5 microcentrifuge column (GE Healthcare). EMCS (final concentration of 2 mM, Dojindo, Kumamoto, Japan) was added to the obtained solution, and the mixture was incubated at r.t. for 1 h. Modified DNA was purified using a Favorprep column according to the reported procedure [28] and eluted with water. Then, the target protein (SA or IgG, approximately 1 mg/mL) and the modified tag DNA were mixed in PBS at equimolar stoichiometry. After incubation at 4 °C overnight, the complex was purified using Amicon Ultra (0.5 mL, molecular weight cutoff of 50 kDa, Merck). Successful labeling was confirmed by SDS-PAGE, using TAMRA fluorescence. 

### 3.3. Photocrosslinking of Model Linker DNA and SA with Tag DNA

SA labeled with tag DNA (final concentration of 1 μM) and biotinylated linker DNA (final concentration of 1 μM) were incubated in PBS at 37 °C for 30 min. For biotin (+) samples, SA was preincubated with 1 mM biotin for 30 min before addition of linker DNA. The samples (10 μL) were irradiated with UV light at 365 nm using a CL-1000 UV Crosslinker (UVP, Upland, CA, USA) for 120 s, before analysis by SDS-PAGE (6% stacking/15% separating gel, 20 mA, 120 min). The gel was imaged with a fluorescein isothiocyanate (FITC) filter set. Precision Plus Dual Color Standard (Bio-rad, Hercules, CA, USA) was used as a protein marker. 

### 3.4. Synthesis of PC-Linker

The linker was synthesized according to the procedure described in our previous paper [31] using biotin and puromycin fragments (Table S1, Supplementary Materials), except that product purification was performed by gel dissection (10% acrylamide gel, 200 V, 30 min; see Figure S4, Supplementary Materials) instead of high-performance liquid chromatography (HPLC). Dissected gel was mashed with BioMasher II (Nippi, Tokyo, Japan), and DNA extraction was performed overnight with Tris-EDTA (TE) buffer. The remaining polyacrylamide was removed by a Spin-X microcentrifuge column (Corning, NY, USA). DNA was ethanol-precipitated using Quick-Precip Plus Solution (Edge Bio, San Jose, CA, USA) and dissolved in water.

### 3.5. Formation of cDNA Display Using PC-Linker

The DNA template (see Figure S5, Supplementary Materials for SBP and our previous paper [30] for BDA) was converted to cDNA display according to the procedure described in our previous paper [16]. When necessary, the template was amplified by PCR using Newleft and cnvK-NewYtag as primers (Table S1, Supplementary Materials). For the cDNA display molecule encoding SBP, biotin (0.1 mM) was added to the buffer of RNase T1, in order to prevent retention of the molecules on SA beads. For confirmation, aliquots of mRNA-linker, input (= mRNA display) and supernatant of SA-bead immobilization, eluant of RNase T1 treatment (= crude cDNA display), supernatant of Ni beads, and finally the collected His tag eluant (= purified cDNA display) were taken and analyzed by SDS-PAGE (4% stacking/6% separating, 20 mA, 120 min) containing 8 M urea. All samples corresponded to 0.5 picomole of library molecules, assuming that every step proceeded with perfect yield. The gel was fluorescently visualized with a FAM filter set, and band intensity was calculated to estimate the cDNA display formation efficiency. Regarding the last three samples, RNase H (Takara, 10 U) and 10× NE buffer 2 (1/10 by volume, NEB, Ipswich, USA) was added to the samples and the mixture was incubated at 37 °C for 30 min before loading onto a gel to digest the RNA/cDNA duplex. 

### 3.6. Photocrosslinking of cDNA Display Molecules and Target Proteins

SA modified with tag DNA (final concentration of 4 μM) and cDNA display encoding SBP (approximately 0.23 picomole) was incubated in PBS (10 μL) at 25 °C for 30 min. For biotin (pre-treatment) samples, 500 μM (final concentration) of biotin was incubated with SA at 25 °C for 30 min before addition of cDNA display. UV irradiation (CL-1000 UV Crosslinker) was then performed for 120 s. For biotin (post-treatment) samples, 500 μM of biotin was added and the samples were incubated at 25 °C for 30 min. All samples were analyzed by native PAGE at 4 °C (8%, 200 V, 150 min for SBP, and 4%, 200 V, 60 min for BDA). For IgG–BDA interaction, essentially similar conditions were used. In this case, instead of biotin, free IgG (40 μM) was added as a competitor. 

### 3.7. Model Selection under Multimolecular Conditions

SA modified with tag DNA (final concentration of 4 μM), cDNA display encoding SBP (approximately 0.14 picomole), cDNA display encoding BDA (approximately 0.25 picomole), and BSA (final concentration of 1%, *w*/*v*) were incubated in PBS (10 μL) at 25 °C for 30 min. UV irradiation (CL-1000 UV Crosslinker) was then performed for 120 s. The samples were analyzed by PAGE (4%, 200 V, 60 min) at 4 °C. As a control, samples without one or more compounds were also prepared and analyzed. Then, the bands corresponding to photocrosslinked product and non-crosslinked cDNA display of lane 5 were dissected and mashed with Biomasher II, and the DNA therein was extracted by overnight incubation with TE buffer. After removing polyacrylamide in a Spin-X microcentrifuge column (Corning), DNA was ethanol-precipitated using Quick Precip-Plus Solution (EdgeBio) and dissolved in water. Then, PCR amplification was performed using T7Ω new and cnvK-NewYtag as primers (Table S1, Supplementary Materials), and the product was analyzed by PAGE (4%, 200 V, 20 min).

## 4. Conclusions

Although in vitro selection of nucleic acid/peptide aptamers based on the SELEX approach was developed about 30 years ago [32,33], almost all the selections so far used solid materials, such as SEPHAROSE columns or magnetic beads, to immobilize target molecules. However, it is well established that this traditional SELEX methodology has several problems, including low resolution (that is, efficacy of separating binder and non-binders), non-specific binding to the solid phase, denaturation of target molecules, and difficulty of eluting strong binders [34,35]. To address these issues, alternative strategies, such as capture SELEX [34] and CE-SELEX [35], which do not rely on immobilized target molecules, were developed for DNA/RNA aptamers. In contrast, much less effort was devoted to peptide aptamers along these lines, except for some examples of cell-based selections [13]. 

Here, we proposed and performed proof-of-concept experiments of a new strategy of in vitro selection using the cDNA display technique, which is (1) solution-based, (2) compatible with multimolecular crowding biosystems, and (3) free of technically challenging control over washing conditions, by taking advantage of covalent photocrosslinking between target molecules and the linker moiety of cDNA display molecules. Another advantage of this method is that the experimenter can precisely and evenly control the concentration of target molecules in the selection system, which is difficult in most solid phase-based selection. 

In this study, we performed PAGE to separate, detect, and collect photocrosslinked products because it is most simple. We note, however, that affinity pull-down using microbeads should also be possible for these purposes, as represented in Figure 1c. One may worry that, if beads are used for pull-down, the problem of non-specific adsorption would not be solved. However, there are several ways to covalently capture target proteins (labeled with specific tags) on beads [36,37,38]; thus, extensive washing (e.g., with SDS and heat) can be performed when necessary.

As far as we know, this is the first study that experimentally demonstrates photocrosslinking between a cDNA display (or mRNA display in a broader sense) and the target proteins. Of course, there are some concerns and drawbacks. Firstly, after photocrosslinking, discrimination between relatively weak and strong binders using the difference of dissociation rate constants [39] (*k*_off_) is not possible. However, this might be solved by adjusting selection conditions. Heterogenous labeling of target proteins with tag DNA is another potential drawback. This could be solved by genetic fusion of tag proteins like SNAP tags [40] and Halo tags [41] to the targets, or by using rare amino acids such as tryptophan for chemical labeling [42], for instance. Introduction of non-natural amino acids in target proteins [43] would also be a promising solution to site-specifically conjugate tag DNAs. We would also like to comment that the position of tag-DNA on the target protein may affect the photocrosslinking efficiency, and detailed experiments will be necessary to investigate this issue in future. 

In short, the photocrosslinking-based selection proposed here should be a promising alternative that may complement traditional SELEX using immobilized target molecules. In principle, this method is applicable to mRNA display in general [10], and we are planning to further improve the methodology to make it more practical. 

## Supplementary Materials

The following are available online at https://www.mdpi.com/1420-3049/25/6/1472/s1: Figures S1 to S8, Table S1.
