# Supplementary material for: Photocrosslinking of cDNA Display Molecules with Their Target Proteins as a New Strategy for Peptide Selection
Source: Molecules. 2020 Mar 24;25(6):1472. doi: 10.3390/molecules25061472 (PMC7146492; doi:10.3390/molecules25061472)
Supplement: Supplementary file 1 [file molecules-25-01472-s001.pdf]

Supplementary Materials

Photocrosslinking of cDNA Display Molecules with Their Target Proteins as a New Strategy for Peptide Selection

Takuya Terai *, Tomoyuki Koike and Naoto Nemoto *

Graduate School of Science and Engineering, Saitama University, 255 Shimo-Okubo, Sakura-ku, Saitama City, Saitama 338-8570, Japan

* Correspondence: tterai@mail.saitama-u.ac.jp, Tel.: +81-48-858-3534 (T.T.); nemoto@fms.saitama-u.ac.jp or nemoto@mail.saitama-u.ac.jp (N.N); Tel.: +81-48-858-3531 (N.N.)

Supplementary Table

Table S1. Primers and synthetic oligonucleotides used in this study.

Name	Sequence (5' to 3')
Newleft	GATCCCGCGAAATTAATACGACTCACTATAGGG
cnvK-NewYtag	TTTCCACGCCGCCCCCCGTCCT
T7Ω new	GATCCCGCGAAATTAATACGACTCACTATAGGGGAAGTAT TTTTACAACAATTACCAACA
biotin fragment	BAA-(rG)-AATTTCCAKGCCGCCCCCCG-(T-NH_2)-CCT
puromycin fragment	(HS)-TCCCCGCGTGCTCFCTC-(Spacer18)$_2$-CCP

Note: F is fluorescein-dT, P is puromycin CPG, Spacer18 is the spacer phosphoramidite 18, B is 5'-biotin-TEG, rG is guanine ribonucleotide, K is ^{cnv}K, SH is terminal thiol, and T-NH_2 is amino-modifier C6 dT (terminology is according to Tsukuba Oligo Service, Japan).

Supplementary Figures

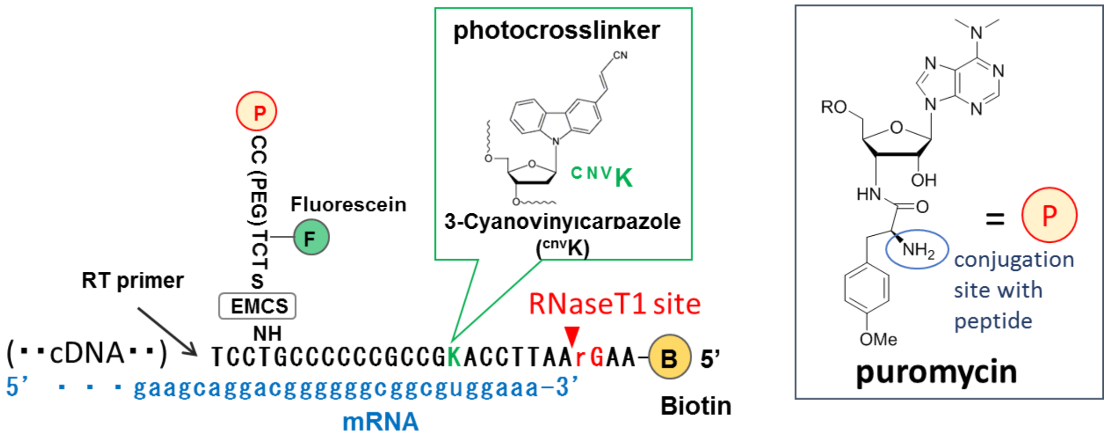

Figure S1. Chemical structure of the current puromycin linker (^{cnv}K rG linker). The linker is basically a branched oligonucleotide, and its functional groups include biotin (for immobilization and purification), fluorescein (for detection), guanine (for cleavage with RNase T1), and reverse transcription (RT) primer, as well as puromycin at the 3' terminal. See reference 16 in the main text for details. For clarity, part of the mRNA sequence is also shown in blue.

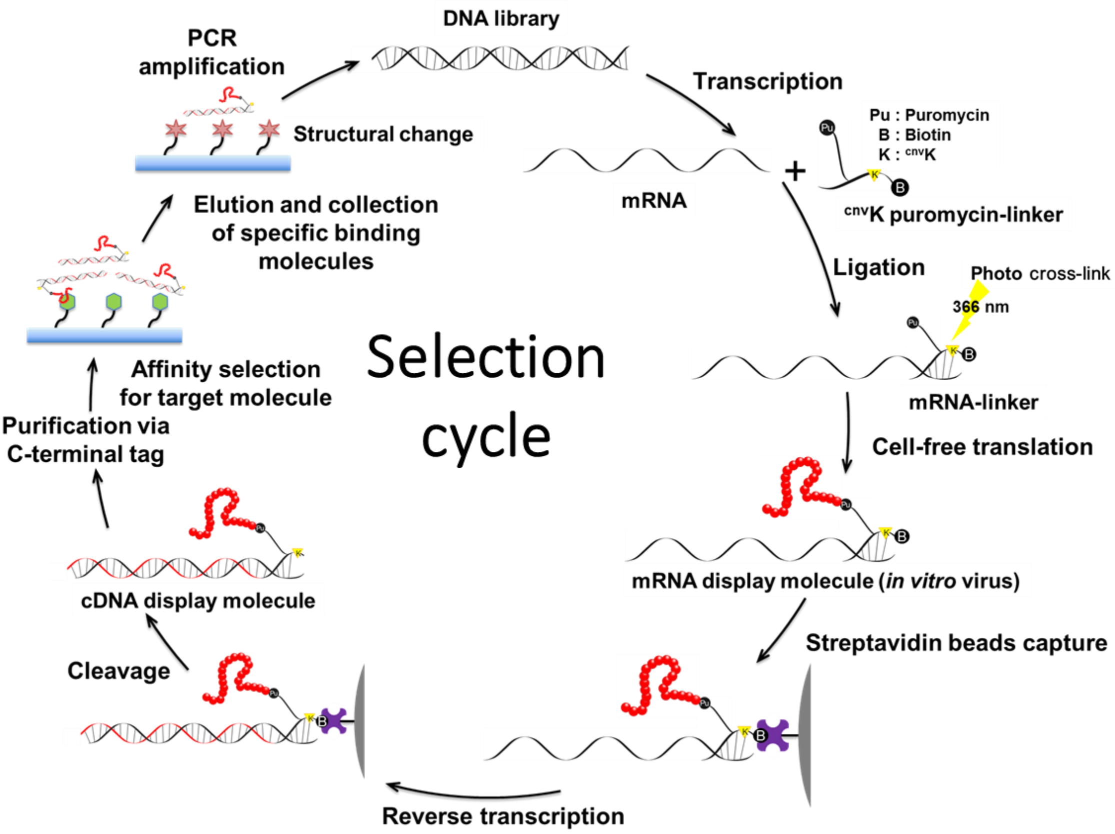

Figure S2. Overview of representative aptamer selection cycle using cDNA display. The DNA library is transcribed to mRNA and ligated to a puromycin linker. After translation *in vitro*, mRNA display molecules (sometimes called *in vitro* viruses) are formed. They are immobilized on beads and reverse transcription is performed. The obtained cDNA display molecules, which are hybridized to mRNA, are cleaved from the beads and purified using a His_6 tag. Then, affinity selection against the target protein is performed and the eluted cDNA is amplified.

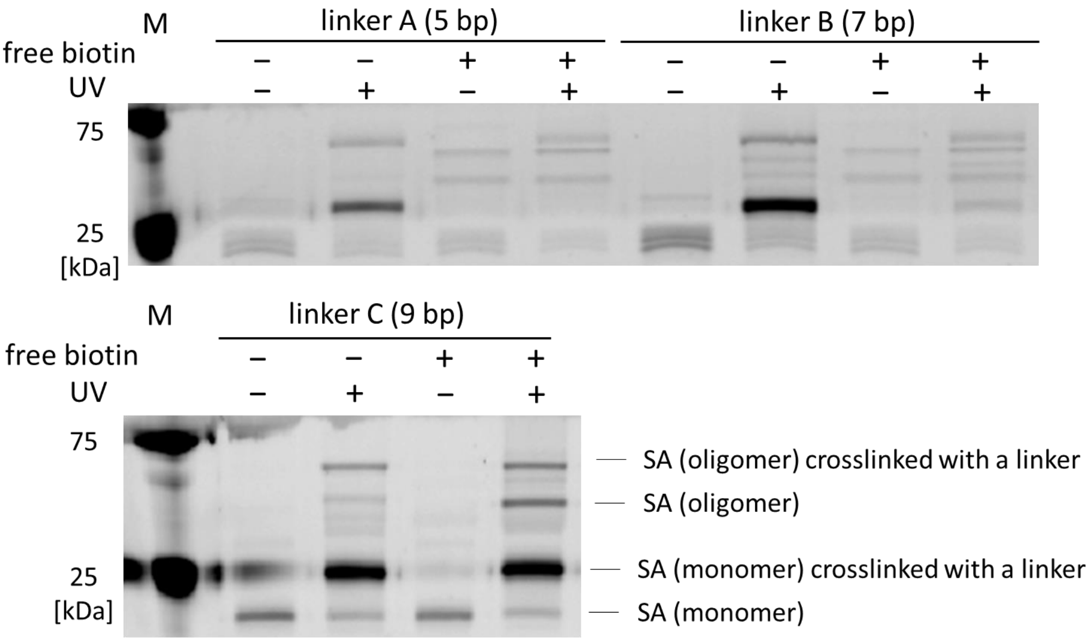

Figure S3. Gel images of photocrosslinked samples. Fluorescence of FAM, which was conjugated to linkers, is visualized. SDS-PAGE was performed at 20 mA for 120 min using 6% stacking/15% separating gel. Free linkers (~6 kDa) are not visible because they eluted out from the gel. After heating and SDS denaturation, most streptavidin (SA) molecules dissociated to monomers, but some of them were retained as oligomers. M indicates fluorescent protein markers. SA molecules that were not crosslinked with a linker are slightly visible under this filter condition because of the partial leakage of TAMRA fluorescence.

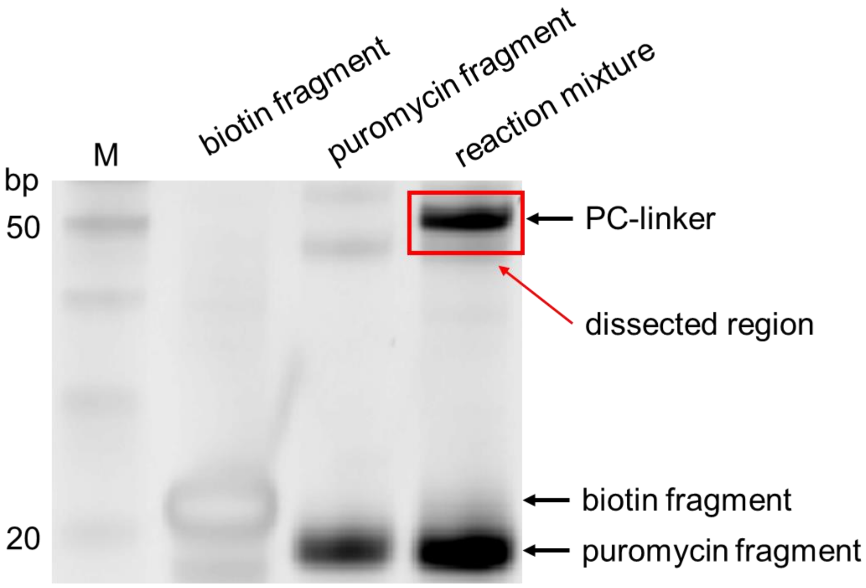

Figure S4. Preparation of the PC-linker. The two modified oligonucleotides (biotin fragment and puromycin fragment, shown in Figure 3) were coupled using EMCS, as described in Section 3. The reaction mixture was analyzed by PAGE (10% gel, 200 V, 30 min) and SYBR Gold staining. For reference, each fragment was also electrophoresed. Note that the signal from the biotin fragment was too strong for quantification in this image. The formed linker was purified by gel dissection and extraction, as described in Section 3.

SBP DNA

14–33	T7
34–36	5`cap
37–107	Ω
110–114	kozak
118–228	SBP
229–240	GGGS
241–258	His_6 Tag
259–267	GGS
268–289	cnvK NewYtag

GATCCCGCGAAATTAATACGACTCACTATAGGGGAAGTATTTTTACAACAATTACCAACA
ACAACAACAAACAACAACAACATTACATTTTACATTCTACAACTACAAGCCACCATGGA
CGAAAAGACGACCGGTTGGAGGGGAGGCCACGTGGTCGAGGGGCTTGCCGGCGAACT
GGAGCAGCTCAGAGCGCGCTTGGAGCACCATCCGCAAGGCCAACGGGAGCCAGGGGG
AGGCAGCCATCATCATCATCATCACGGCGGAAGCAGGACGGGGGGCGGCGTGGAAAGA
ATTC

Figure S5. DNA sequence of the construct encoding SA binding peptide (SBP). The DNA was previously synthesized from fragments using primer extension PCR in our laboratory.

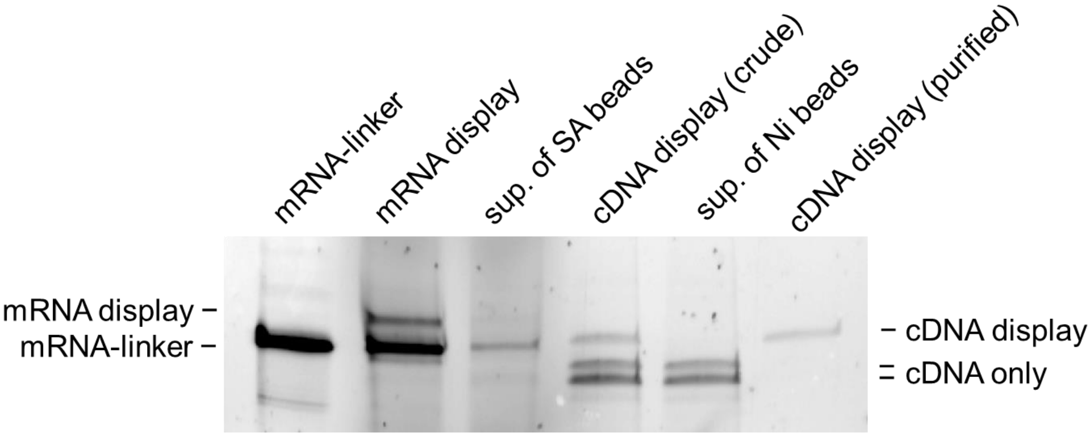

Figure S6. Confirmation of cDNA display formation using the new PC-linker. The mRNA-linker complex encoding SBP (lane 1) was converted to an mRNA display (lane 2), and immobilized on SA beads (supernatant was applied in lane 3). After reverse transcription and elution from beads (lane 4), the cDNA display molecules were purified by Ni beads using His_6 tags (supernatant and eluant were applied to lanes 5 and 6). All samples corresponded to 0.5 picomole if all the chemical reactions proceeded to completion. The efficiency of cDNA display formation was calculated to be 10% using a comparison of the band intensities of lanes 1 and 6. The efficiency was not significantly different from that of a previous linker (data not shown).

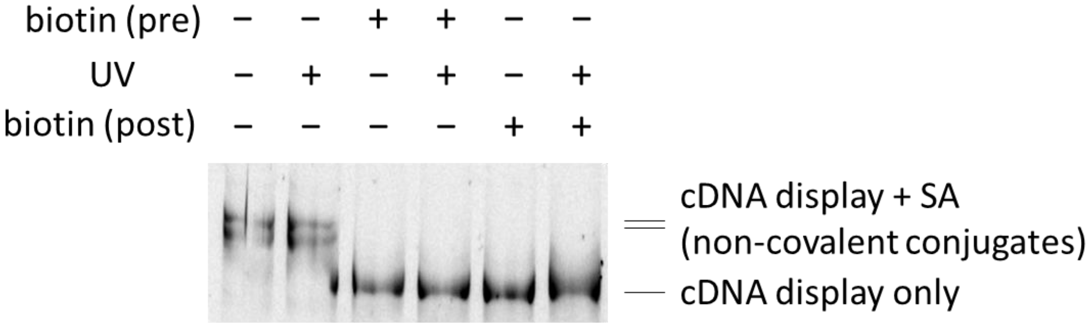

Figure S7. Control experiment of crosslinking. cDNA display encoding SBP and intact SA (i.e. SA without a DNA tag) were incubated with and without biotin, irradiated with UV, and analyzed by PAGE. For lanes 5 and 6, biotin was added after irradiation. The gel was visualized with FAM attached to cDNA display molecules. In all cases, no difference was observed between irradiated and non-irradiated samples.

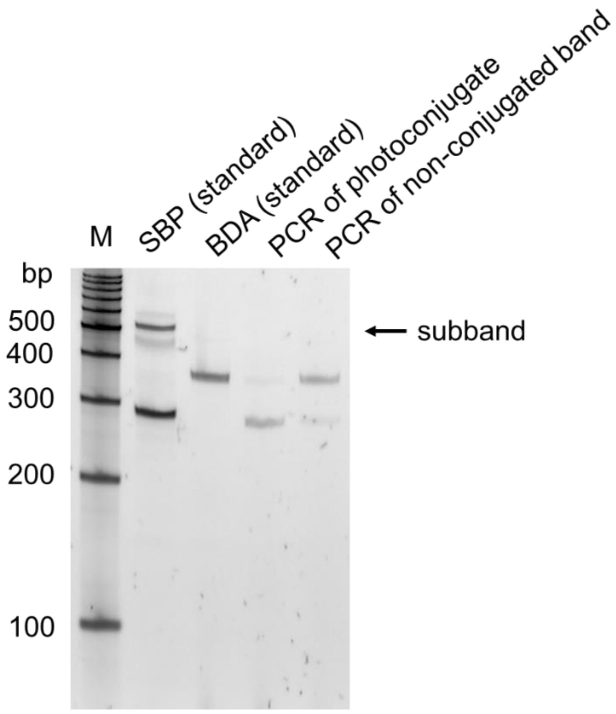

Figure S8. PCR amplification of photocrosslinked products. The bands corresponding to photocrosslinked products and non-crosslinked cDNA display molecules (see lane 5 of Figure 5) were dissected and the DNA they contained were amplified by PCR. The gel was stained with SYBR Gold. As expected, DNA encoding a binding peptide (SBP) was selectively recovered over a non-binding peptide (BDA).
